# Geobiochemistry characteristics of rare earth elements in soil and ground water: a case study in Baotou, China

**DOI:** 10.1038/s41598-020-68661-4

**Published:** 2020-07-16

**Authors:** Shuting Tang, Chunli Zheng, Minjie Chen, Weiqi Du, Xin Xu

**Affiliations:** 1 School of Energy and Environment, Inner Mongolia University of Science and Technology, Baotou, 014010 People’s Republic of China; 2 School of Life Science and Technology, Inner Mongolia University of Science and Technology, Baotou, 014010 People’s Republic of China

**Keywords:** Biogeochemistry, Ecology, Environmental sciences

## Abstract

The distribution of rare earth elements and the microbial community in nearby ground water and soil were influenced by tailings ponds. Accordingly, the behaviors of rare earth elements in ground water and soil around the tailings pond, and the changes of microbial communities were both investigated in this study. The results showed that rare earth elements accumulated in ground water and soil around the tailings pond appeared as light rare earth elements enrichment. Through the normalization of rare earth elements, different extents of anomaly (from negative to positive) were observed for Ce and Eu in the distribution patterns of REEs in groundwater, however, Ce and Eu were negatively anomaly in soil. According to the correlation analysis, Mn^2+^, SO_4_^2−^, Cl^−^, ammonia nitrogen and Ca^2+^ are significantly correlated with the distribution of rare earth elements. Meanwhile, there were the same dominant bacteria in ground water and soil including *Actinobateria*, *Proteobacteria* and *Acidobacteria* at the phylum level. This microbial community composition is similar to that reported in arid lands around the world. On the other hand, *Bacillus* and *Blastococcus* showed significant correlation with rare earth elements at the genus level. This study might provide an important basis for the risk assessment of REEs in the environment.

## Introduction

Rare earth elements (REEs) are composed of fifteen lanthanides from lanthanum (La, Z = 57) to lutetium (Lu, Z = 71) or, according to International Union of Pure and Applied Chemistry (IUPAC), even yttrium (Y, Z = 39) and scandium (Sc, Z = 21). Sc and Y are considered REEs because they exhibit similar properties to the lanthanide family. Remarkably, Pm is the only element that does not form stable isotopes. It is the product of natural fracture processes and its total concentration in the crust does not exceed 600 g^1^. Based on the chemical, physical and geochemical properties of lanthanides, they are usually divided into two groups: LREEs from La to europium (Eu, Z = 92) and HREEs from gadolinium (Gd, Z = 64) to Lu. LREEs have lower atomic numbers, larger ionic radii, higher solubility and alkalinity, while HREEs are sparingly soluble elements with higher atomic numbers, smaller ionic radii and lower alkalinity. Lanthanides exist in the form of trivalent ions (Ln^3+^) with the exception of cerium (Ce, Z = 58) and Eu that can additionally exist as tetravalent and divalent ions, respectively^2^. At present, REEs are indispensable in many industries^3^. Before considering geopolitical and economic factors, little attention has been paid to the environmental hazards associated with these REEs. Their increasing use in industry has led to an increase in release points into the environment and raised the prospect of REEs as important environmental pollutants^4^.

As reported, Asia has 14 rare earth producers, including China, Vietnam and India and so on; Europe has six; Australia is rich in rare earths, while the United States and Canada have many small reserves^5^. About 97% of the global supply of rare earths comes from China, especially from the Fe-REE-Nb mineral deposit at Bayan Obo in Inner Mongolia, it holds 36% of the world's rare earth reserves, which total about 520 million tons^6^. While the harmful of REEs are widespread, including on the earth's environment, aquatic life and human life. For instance, high concentrations of lanthanides can cause slow growth and other negative effects in animals^7^. Cheng et al.^8^ reported that after 20.00 mg/kg lanthanide elements added into the rat diet, the cell structure and liver function were impaired, the level of reticular cells in the blood decreased, and the growth was inhibited. While REEs' data is scarce in the food chain, there are concerns about potential human health problems. REEs could be accumulated in the brain ranging from 0.10 to 19.40 μg/g and in human rib bones from 0.40 to 22.00 μg/kg^9,10^. In fact, in one report, high levels of REEs were detected in hair from the scalp of children living in a rare earth metals mining area in China^11^. De-la-Iglesia-Iñigo et al.^12^ reported that REEs could cause red blood cell abnormality, its microcytosis rate up to 25%, anemia up to 10%, hemoglobin disease up to 12%. All the facts are presented to emphasize that the dangers of REEs, it is necessary to study the behavior of REEs in the environment under the influence of human factors.

Microorganisms in natural environment are quite important in maintaining soil biological activity, which are likely to lessen the pollutants levels^13,14^. It has been recognized that REEs have the Hormesis effect on the growth of microorganisms. Moriwaki et al.^15^ reported that the microbes were capable of adsorb the rare earth ions was given priority to with bacteria. In particular, the REEs partitioning between bacteria and fluid phases leads to a typical REEs signature^16^. In this context, microbial activity may play a key role in the sample points, influencing the distribution of trace elements onto microbial surfaces.

Based on this, taking a rare earths tailing pond in northern China as the research area, the surrounding soil and ground water used as the research object were selected, and discussed the behavior of rare earths among them. The overall objective of this study is to present behaviors of 14 REEs in soil and ground water from anthropogenic sources and find the dominant microbiome around the tailings pond, and further estimate the interaction of the content of REEs with microbial community around the tailings pond. Therefore, this study will provide necessary guidance for the future risk assessment of REEs.

## Materials and methods

### Site description

**Figure 1:**
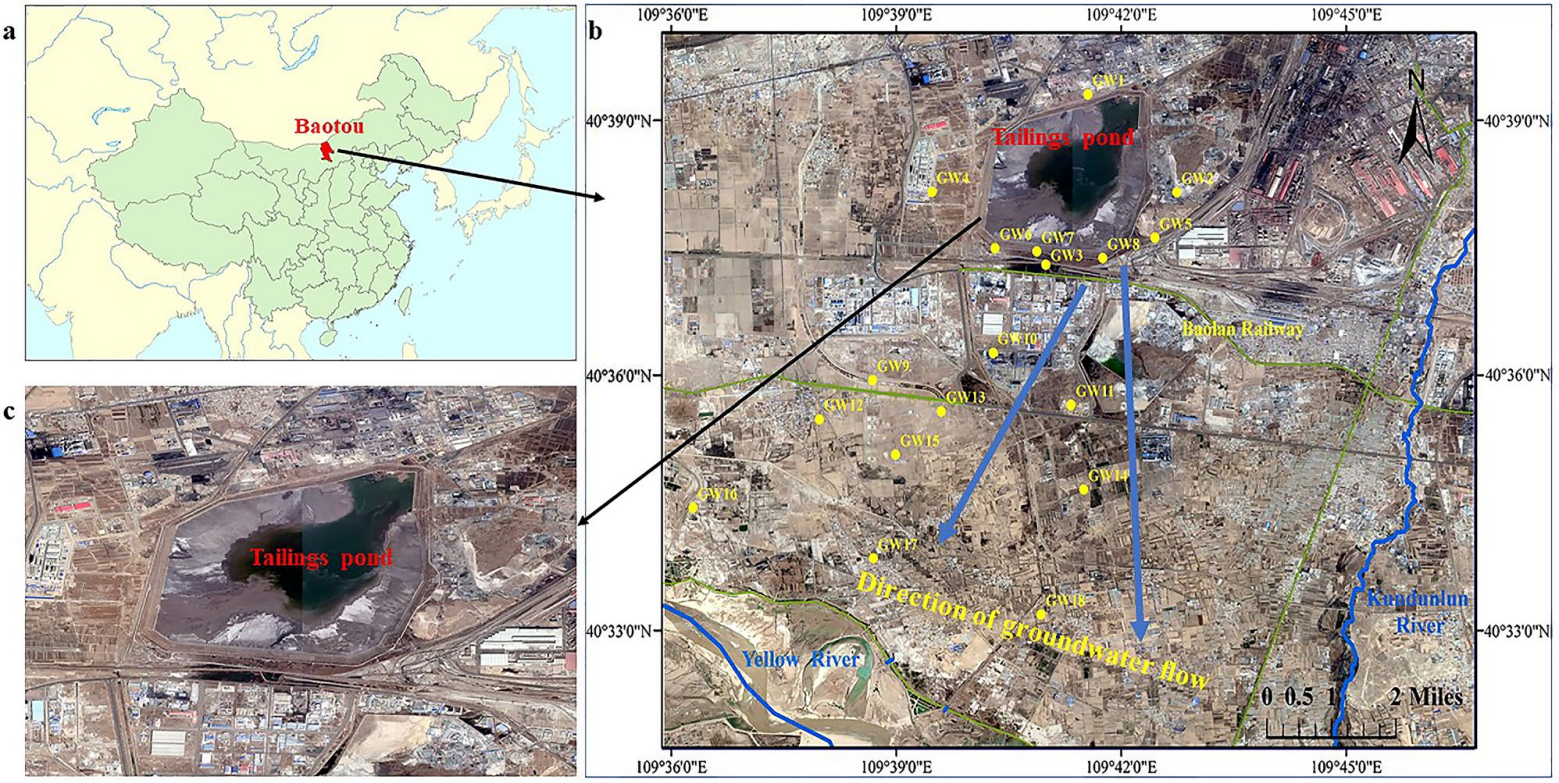
Location of the study site in China (**a**); the Bayan Obo tailings impoundment and sampling site (**b**); and a larger view of the impoundment (**c**). Google Earth version 7.1.8.3036 (https://download.pchome.net/industry/geography/detail-20351.html) was used to prepare a map showing the locations of sampling in ArcGIS version 10.2.0 (https://www.esri.com/en-us/home).

The Baotou REEs tailings pond (40°30′ ~ 40°42′N, 109°33′ ~ 109°55′E) is located at 12 km distance from the west of Baotou City (Fig. 1). The average wind speed was 2.80 m/s, and maximum wind speed was 16.30 m/s of the study region. The highly concentrated cold air in the western Inner Mongolia Plateau caused dust weather in Baotou under appropriate atmospheric circulation conditions, which occurred 9 times a year on average. The soil type in the study area was chestnut soil. The pond has a 13.60 km perimeters and its tailings impoundment covers an area of 12 km^2^, about 3.50 km long from south to north and 3.20 km from east to west. The remaining slurry is stored in Baotou rare earth tailings reservoir after the Bayan Obo rare earth ore is processed and smelted. Over the past 30 years, the REEs industry in Baotou has rapidly grown, but lack of effective pollution control has resulted in REEs expansion and accumulation. The rare earth tailings pond is open to the environment, which damages the environment around the tailings pond and becomes the source of man-made pollution.

### Field sampling and pretreatment

There were eighteen sampling sites in the south and southwest of the tailings pond, as shown in Fig. 1. Eighteen groundwater samples were collected from drilled monitoring wells at an average depth of 30 m. All water samples were filtered (0.45 μm, regenerated cellulose) and then stored at 4 °C in darkness prior to analysis. Filtered (0.22 μm, regenerated cellulose) part of the water sample from GW1, GW3, GW10, GW13, GW15 and GW17, and stored the filter film with the filter material in the refrigerator at − 80 °C for DNA extraction.

In total, eighteen soil sampling sites nearby eighteen ground water samples were selected for chemical analysis. About 2 kg of 20 cm, 40 cm and 60 cm soil were taken at points GW1 ~ GW18, and then mixed into sterile self-sealing bags, and stored at 4 °C for subsequent analysis, marked as S1 to S18. Select partial soil samples at S1, S8, S10, S11, S13, S14 and S15 were stored − 80 °C before DNA extraction.

### Chemical analysis

REEs measured in this study include La, Ce, Pr, Nd, Sm, Eu, Gd, Tb, Dy, Ho, Er, Tm, Yb and Lu. Sc was excluded due to known analytical interference with ICP-MS analysis^17^. All REEs and Fe analyses were conducted at the Nuclear Industry Geological Institute of the Beijing Analysis Research Center by inductively-coupled plasma mass spectrometry (ICP-MS). Filtered water samples were preserved with HNO_3_ (2%) and soil samples were digested with HNO_3_-HF-HClO_4_ before analyzed. Anions, such as Cl^−^, F^−^, NO_3_^−^, SO_4_^2−^ and HCO_3_^−^ were measured by ion chromatograph (Dionex-500, precision ± 5–10%). The content of K^+^, Ca^2+^, Na^+^, Mg^2+^ and Mn^2+^ were determined by using a flame atomic absorption spectrophotometer (PE-AA800). Besides, ammonia nitrogen in water and wastewater by nessler's Reagent Spectrophotometry. All analyses were performed as triplicates and measurement errors showed to be ≤ 5% for all samples.

### DNA extraction, PCR amplification and Illumina MiSeq sequencing

Soil samples used for DNA extraction were extracted from 0.5 g soil and DNA was extracted according to manufacturer's agreement using the FastDNA rotation kit (Mp Biomedicals, Illkirch, France). The DNA extract was checked on 1% agarose gel, DNA concentration and purity were determined with NanoDrop 2000 UV–vis spectrophotometer (Thermo Scientific, Wilmington, USA). The hypervariable region V3-V4 of the bacterial 16S rRNA gene were amplified with primer pairs 338F (5′-ACTCCTACGGGAGGCAGCAG-3′) and 806R (5′-GGACTACHVGGGTWTCTAAT-3′) by an ABI GeneAmp 9,700 PCR thermocycler (ABI, CA, USA). The PCR amplification of 16S rRNA gene was performed as follows: initial denaturation at 95 °C for 3 min; 30 cycles of denaturation at 95 °C for 30 s, primer annealing at 55 °C for 30 s, and extension at 72 °C for 45 s, followed by a final extension period of 10 min at 72 °C. For each sample, all three soil replicates were independently analyzed and averaged. Purified amplicons were pooled in equimolar and paired-end sequenced (2 × 300) on an Illumina MiSeq platform (Illumina, San Diego, USA) according to the standard protocols by Majorbio Bio-Pharm Technology Co. Ltd. (Shanghai, China)^18,19^.

### Statistical analysis

Total REEs concentrations (ΣREE) were calculated as the sum of the concentrations of each individual REEs. In the text, we refer to heavy and light REEs. HREEs include Gd, Tb, Dy, Ho, Er, Tm, Yb, Lu; LREEs include La, Ce, Pr, Nd, Pm, Sm, Eu^3^, although the specific elements in each group vary between studies. At the same time, in order to eliminate the characteristic zigzag distribution pattern of REEs and to identify the individual REEs anomalies, measured concentrations of REEs were normally normalized. The method eliminates variations in abundance between lanthanide elements with even and odd atomic numbers, which can determine fractionation between these elements. This study applied chondrite-normalized^3,20^. The following formula was utilized to calculate the value of positive and negative anomalies (Eu and Ce) of rare earth elements:

1 $$\delta Ce=\frac{{\left[Ce\right]}_{N}}{{\left({\left[La\right]}_{N}\times {\left[Pr\right]}_{N}\right)}^{0.5}}$$

2 $$\delta Eu=\frac{{\left[Eu\right]}_{N}}{{\left({\left[Sm\right]}_{N}\times {\left[Gd\right]}_{N}\right)}^{0.5}}$$

where $\delta Ce$ and $\delta Eu$ represent the anomalies of Ce and Eu respectively, values > 1 indicate positive anomalies and values < 1 indicate negative anomalies. Ce_N_, Eu_N_, La_N_, Pr_N_, Sm_N_, and Gd_N_ are normalized values against chondrite. Statistical analyses were performed using SPSS version 19.0 (SPSS Inc., Chicago, IL, USA).

## Results

### Distribution characteristics of REEs in ground water

In this study, ground water samples were collected from 18 ground water monitoring wells around tailings ponds and their chemical characteristics were also having been determined, as showed in Figure S1. Fe, Mn^2+^, Cl^−^, SO_4_^2−^, ammonia nitrogen and total hardness showed the same trend and decreased with distance. The ground water environmental quality standard (III Grade, National Standard Bureau of PR China, GB3838-2002, the water quality above III Grade can be used for living and drinking after treatment, but the water quality below III Grade was bad and cannot be used as drinking water source) was used as the evaluation standard. The ratio of the number of wells with Fe, Mn^2+^, Cl^−^, SO_4_^2−^, ammonia nitrogen and total hardness exceeding the standard in the total number of wells was 33.33%, 61.11%, 66.67%, 77.78%, 100% and 81.25%, respectively.

**Figure 2:**
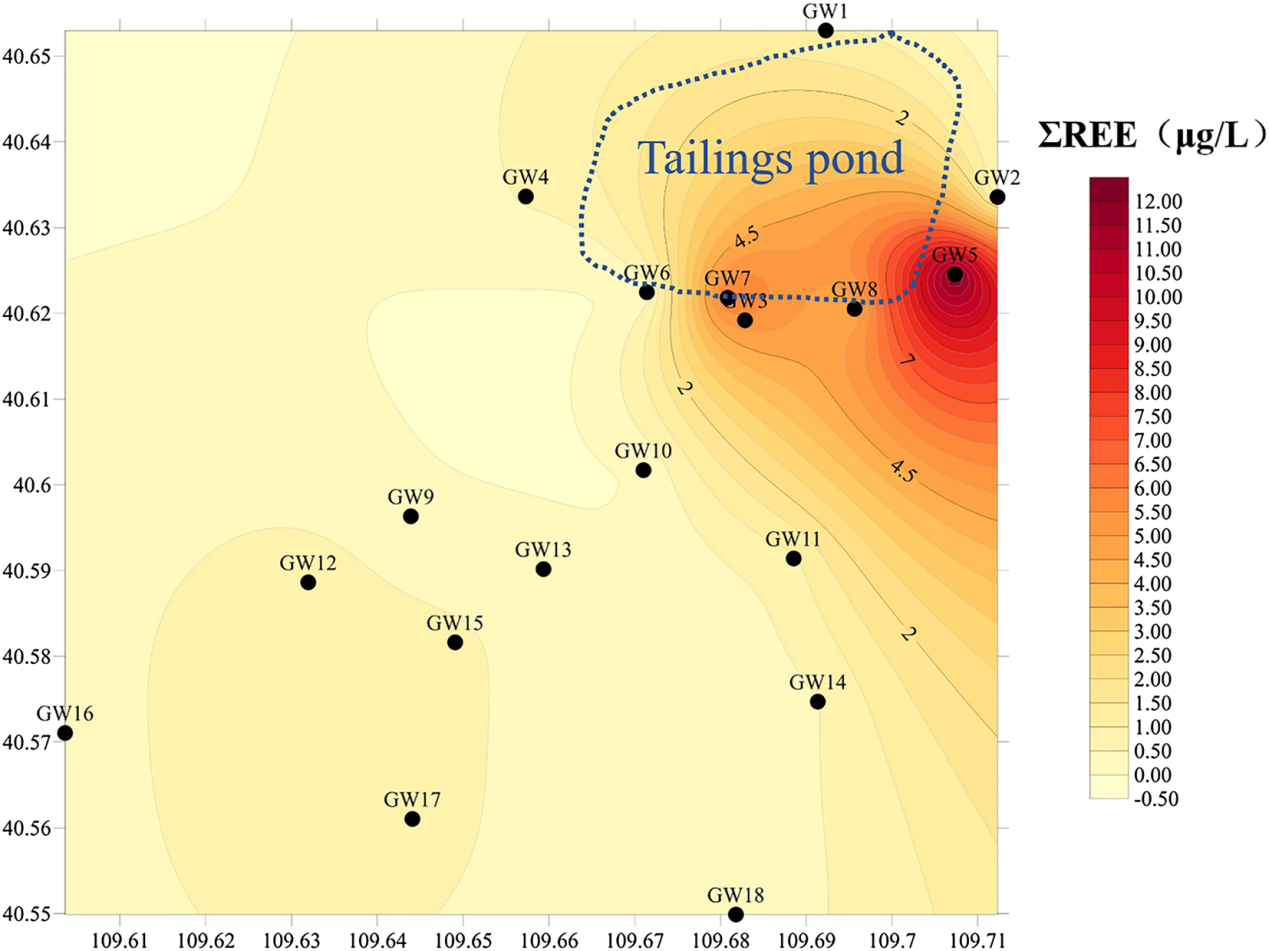
Distribution of rare earth elements in the ground water surrounding the rare earth tailings pond (μg/L).

In order to study the accumulation of REEs in ground water, the concentration of REEs in 18 ground water samples around the tailings pond were measured. The total REEs concentrations in ground water ranged from 0.0820 to 12.3 μg/L, and rare earth in the ground water accumulated in the southeast of the tailings pond (Fig. 2). In addition, the concentrations of REEs in ground water around the tailings pond decreased in the order of Ce > La > Nd > Pr > Gd > Sm > Dy > Er > Eu > Yb > Tb > Ho > Tm > Lu. Chondrite-normalized REEs patterns for ground waters around the tailings were shown in Fig. 4b and Table 1. The well points have the same normalization pattern with a predominance of LREEs over HREEs.

**Table 1:** Distribution characteristics of REEs in ground water surrounding tailings pond.

Samples	δCe	δEu	LREEs	HREEs	R_(L/H)_	(La/Yb)_N_	(La/Sm)_N_
GW1	0.9143	0.9471	0.941	0.0940	10.0	11.98	2.169
GW2	1.065	2.038	0.579	0.0890	6.51	5.099	2.537
GW3	1.105	1.597	4.86	0.474	10.3	9.458	4.320
GW4	1.149	1.405	0.491	0.0790	6.22	4.527	1.478
GW5	0.9849	0.6766	11.8	0.457	25.9	50.67	5.216
GW6	0.6682	1.411	0.130	0.0470	2.77	1.445	0.8205
GW7	1.074	0.7295	5.97	0.291	20.5	38.00	4.216
GW8	1.128	0.8380	4.23	0.330	12.8	15.28	4.150
GW9	0.6414	13.70	0.125	0.0250	5.00	3.708	1.258
GW10	0.7369	6.784	0.0670	0.0150	4.47	2.697	0.5806
GW11	1.168	1.184	0.765	0.138	5.54	4.787	1.654
GW12	1.103	8.995	0.703	0.0350	20.1	24.95	3.581
GW13	0.8345	11.86	0.224	0.0330	6.79	8.090	1.258
GW14	1.120	2.231	0.419	0.0670	6.25	5.455	3.499
GW15	1.497	4.842	0.545	0.0440	12.4	7.146	1.150
GW16	0.8595	1.934	0.276	0.0970	2.85	2.809	1.048
GW17	1.312	1.241	0.555	0.102	5.44	7.791	1.677
GW18	1.026	2.293	0.133	0.0290	4.59	2.966	0.7688

*LREEs* total LREEs (La to Gd), *HREEs* total HREEs (Tb to Lu), (La/Yb)_N_ and (La/Sm)_N_ are normalized concentration ratios in the samples; δCe is the value of the Ce anomaly calculated by δCe = [(Ce_N_)/((La_N_*Pr_N_)^1/2^)], δEu is the value of the Eu anomaly calculated δEu = [(Eu_N_)/((Sm_N_*Gd_N_) ^1/2^)]; Ce_N_, Eu_N_, La_N_, Pr_N_, Sm_N_, and Gd_N_ are normalized values against chondrite; R_(L/H)_ = LREEs/HREEs.

The distribution patterns of REEs in ground water were characterized by obvious fractionation of LREEs and HREEs with the LREEs/HREEs ratios of 2.77 ~ 25.9, and (La/Yb)_N_ of 1.445 ~ 50.67. The degree of LREEs fractionation with (La/Sm)_N_ of 0.5806 ~ 5.216. Most sampling points presented the positive anomaly of Ce and Eu, however, GW1, GW5, GW6, GW9, GW10, GW13 and GW6 were negative anomalies of Ce, while GW1, GW5, GW7 and GW8 were negative anomalies of Eu. Individual anomalies showed differentiation between selected elements (Ce and Eu) and the other REEs (Table 1).

Baotou environmental monitoring station, Inner Mongolia, China detected ground water leakage around the pond, and various degrees of ground water pollution were found with relatively lower metals concentration and higher anionic concentration^21–23^. Therefore, in addition to REEs, for our ground water correlation analysis we chose to also look at Fe, Mn^2+^, Cl^−^, SO_4_^2−^, ammonia nitrogen and some other ions (HCO_3_^−^, total hardness). Correlation analysis showed that total hardness (r = 0.541, *p* < 0.05), Mn^2+^ (r = 0.608, *p* < 0.01) and ammonia nitrogen (r = 0.626, *p* < 0.01) were significantly positively correlated with the ΣREE. However, there was no significant correlation between pH, Fe, Cl^−^, SO_4_^2−^, HCO_3_^−^ and ΣREE. δCe was not related to physical–chemical indexes, but δEu was significantly negatively correlated with the Cl^−^ (r = − 0.505, *p* < 0.05), SO_4_^2−^ (r = − 0.559, *p* < 0.05) and total hardness (r = − 0.483, *p* < 0.05), but showed no correlation to other physical–chemical indexes (Table 3).

### Distribution characteristics of REEs in soil

In soil, the pH value of soil varied from 8.00 to 8.87, with an average value of 8.53. Therefore, the soil samples were all alkaline. The variation ranges of SO_4_^2−^ and Cl^−^ content in the soil was 394.120 ~ 2007.73 mg/L and 50.250 ~ 586.70 mg/L, with an average value of 765.42 mg/L and 193.42 mg/L, respectively. Generally, they decreased with the increase of the distance from the tailings pond. The contents of Na^+^, K^+^, Ca^2+^ and Mg^2+^ ranged from 103.49 to 219.73 mg/L, 35.890 to 118.99 mg/L, 140.62 to 474.85 mg/L, and 40.150 to 155.16 mg/L, respectively. The mean values were 137.24 mg/L, 72.120 mg/L, 244.56 mg/L, and 66.000 mg/L. The contents of Na^+^, K^+^, Ca^2+^ and Mg^2+^ showed no significant change trends (Figure S2).

**Figure 3:**
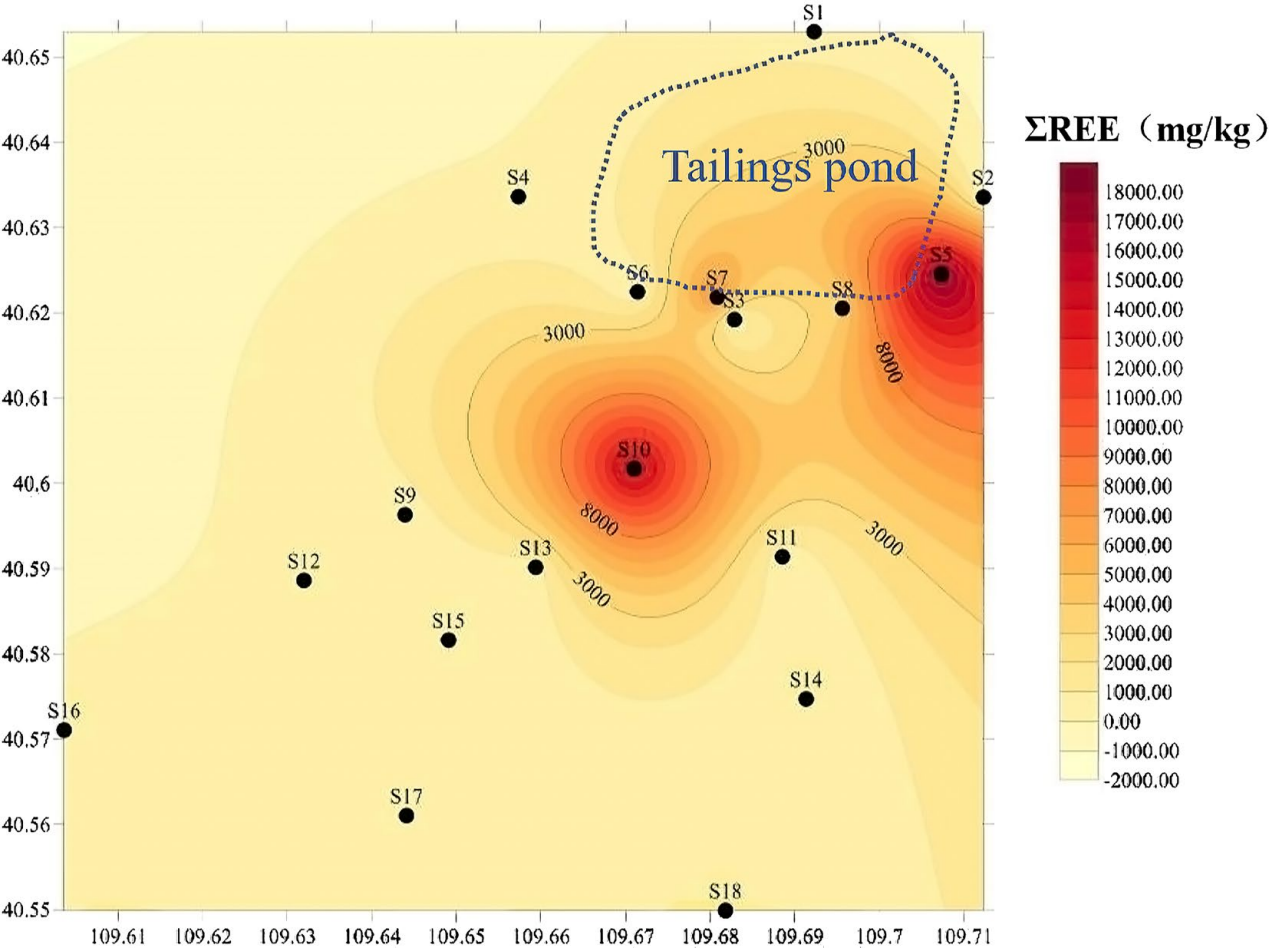
Concentrations of rare earth elements in the soil surrounding the rare earth tailings pond (mg/kg).

The contents of REEs in the soil of most sampling sites around tailings ponds were significantly higher than the geometric average values in Inner Mongolia. The total amount of REEs in the soil around the tailings pond ranges from 157.580 to 18,543.4 mg/kg, with an average of 2,769.95 mg/kg, The soil around the rare earth tailings pond has accumulated a lot of REEs, the content of REEs was higher the closer to the tailings pond. REEs accumulated in the south and southeast of the tailings pond (Fig. 3).

**Figure 4:**
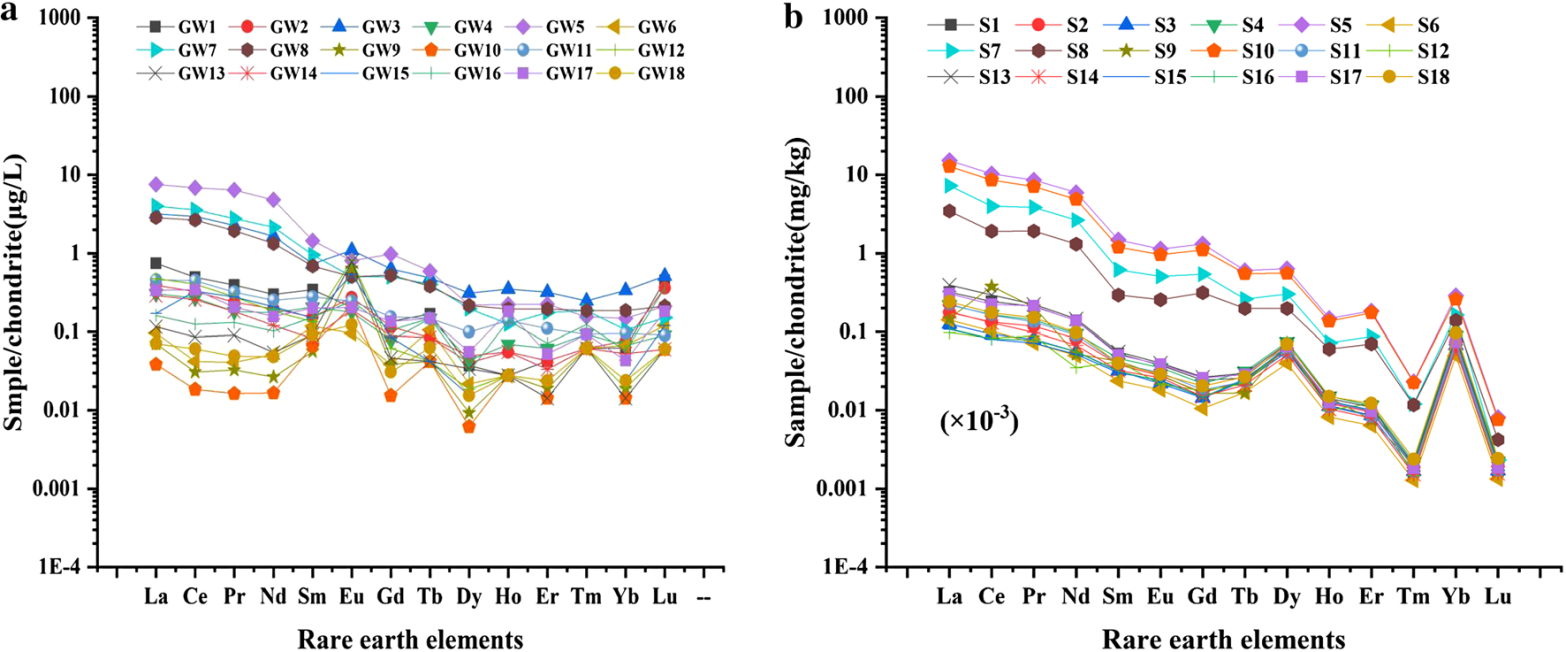
Chondrite normalized patterns of REEs concentrations in ground water (**a**) and soil (**b**) around tailings pond. REEs in ground water and soil around the tailings pond all performance as Light rare earth elements enrichment.

According to the standardized distribution pattern diagram of chondrite of REEs in soil (Fig. 4b), it could be seen that the soil around the tailings pond was rich in LREEs, and at the same time, normalized REEs pattern also showed that the content of REEs was higher the closer to the tailings pond (such as the normalized curve of point GW5, GW7 and GW8 near the tailings pond was slightly higher than other well points). And after the normalization treatment, the range of LREEs/HREEs, (La/Yb)_N_, (La/Sm)_N_ in the soil samples were 7.350 ~ 31.96, 7.070 ~ 332.1 and 2.306 ~ 11.83 respectively, all more than 1, therefore the samples are relatively enriched with LREEs than HREEs, which was consistent with the observation results of the standardized distribution pattern diagram of chondrites of REEs in the soil (Table 2). According to the calculation formula of positive and negative outliers of Eu and Ce, it was concluded that Eu presented negative anomaly, and a slightly negative Ce anomaly (except for S6 and S9) indicated that Ce, Eu were a slight deficit in soil (Table 3).

**Table 2:** Distribution characteristics of REEs in soil surrounding tailings pond.

Samples	δCe	δEu	LREEs	HREEs	R_(L/H)_	(La/Yb)_N_	(La/Sm)_N_
S1	0.9390	0.6091	415.69	18.970	21.91	30.63	6.022
S2	0.9201	0.6268	233.69	17.320	13.49	14.50	4.575
S3	0.9275	0.5900	165.25	15.600	10.59	11.74	3.944
S4	0.9381	0.6380	285.40	21.540	13.25	15.63	4.763
S5	0.9035	0.4719	17,981	562.59	31.96	332.1	10.27
S6	1.013	0.6673	170.71	11.360	15.03	17.79	5.912
S7	0.7539	0.5078	7,675.0	240.80	31.87	279.3	11.82
S8	0.7415	0.4876	3,703.1	152.40	24.30	156.1	11.83
S9	2.179	0.6147	416.32	17.710	23.51	10.89	3.445
S10	0.9018	0.4830	15,005	484.54	30.97	310.6	10.79
S11	0.9366	0.6473	280.15	16.630	16.85	20.99	5.630
S12	0.9019	0.4535	138.71	18.870	7.350	7.070	2.306
S13	0.9879	0.6039	485.21	22.250	21.81	29.59	7.041
S14	0.9802	0.6886	220.33	14.740	14.95	18.58	5.540
S15	0.9121	0.6629	145.04	17.320	8.370	8.410	3.544
S16	0.8738	0.6382	149.42	16.980	8.800	8.770	3.485
S17	0.8784	0.6132	395.79	20.690	19.13	26.03	5.975
S18	0.9160	0.5850	301.85	21.090	14.31	15.62	6.032

**Table 3:** Correlation analysis between ΣREE, δCe, δEu and chemical indexes in ground water.

	pH	Fe	Mn^2+^	Cl^−^	SO_4_^2−^	HCO_3_^−^	Ammonia nitrogen	Total hardness
ΣREE	− 0.311	0.209	0.608**	0.090	0.462	− 0.263	0.626**	0.541*
La	− 0.315	0.217	0.621**	0.090	0.463	− 0.269	0.626**	0.545*
Ce	− 0.310	0.206	0.606**	0.079	0.452	− 0.264	0.614**	0.531*
δCe	0.202	− 0.010	− 0.015	0.061	0.012	0.147	− 0.100	− 0.059
δEu	0.319	− 0.392	− 0.374	− 0.505*	− 0.559*	− 0.050	− 0.129	− 0.483*

**Significant correlation at the 0.01 level (bilateral); *Significant correlation at the 0.05 level (bilateral).

Correlation analysis was conducted between REEs and various chemical properties in soil, as showed in Table 4. The result indicated that REEs and Ca^2+^, SO_4_^2−^ were significantly positively correlated (r = 0.621, 0.807, *p* < 0.01), furthermore, REEs showed positive correlation with Cl^−^ (r = 0.541, *p* < 0.05). Na^+^, K^+^, Mg^2+^ were negatively correlated with La, Ce and ΣREE (r_1_ = − 0.141, − 0.141, − 0.141; r_2_ = − 0.364, − 0.360, − 0.365; r_3_ = − 0.255, − 0.247, − 0.251; *p* < 0.05), however, it did not reach a significant level. δCe also was not related to chemical indexes, but δEu was significantly negatively correlated with the Ca^2+^ (r = − 0.637, *p* < 0.01), SO_4_^2−^ (r = − 0.658, *p* < 0.01) respectively.

**Table 4:** Correlation analysis between ΣREE, δCe, δEu and chemical indexes in soil.

	pH	Na^+^	K^+^	Ca^2+^	Mg^2+^	SO_4_^2−^	Cl^−^
ΣREE	0.152	− 0.142	− 0.365	0.621**	− 0.251	0.807**	0.541*
La	0.148	− 0.141	− 0.364	0.620**	− 0.255	0.822**	0.524*
Ce	0.156	− 0.141	− 0.360	0.616**	− 0.247	0.793**	0.551*
δCe	− 0.018	0.077	0.214	− 0.156	0.028	− 0.260	0.061
δEu	0.051	0.319	0.361	− 0.637**	0.211	− 0.658**	− 0.256

**Significant correlation at the 0.01 level (bilateral); *Significant correlation at the 0.05 level (bilateral).

### Microbial community diversity in typical ground water and soil samples around tailings ponds

On account of the concentration of REEs in soil and ground water decreased gradually in the south side of the tailings pond along the direction of ground water flow. Therefore, this study selected the ground water and soil samples in this direction for the analysis of microbial community. A total of 229,802 and 477,145 high-quality bacterial 16S rRNA gene sequences were obtained from six ground water samples (GW1, GW3, GW10, GW13, GW15, GW17) and seven soil samples (S1, S8, S10, S11, S13, S14, S15), respectively. The Shannon index representing the bacterial alpha diversity were shown in Table S1. The higher the Shannon index, the higher the biodiversity. The change of bacterial community diversity in soil and groundwater was not significant, but it tended to higher with the increase of distance (except GW17).

**Figure 5:**
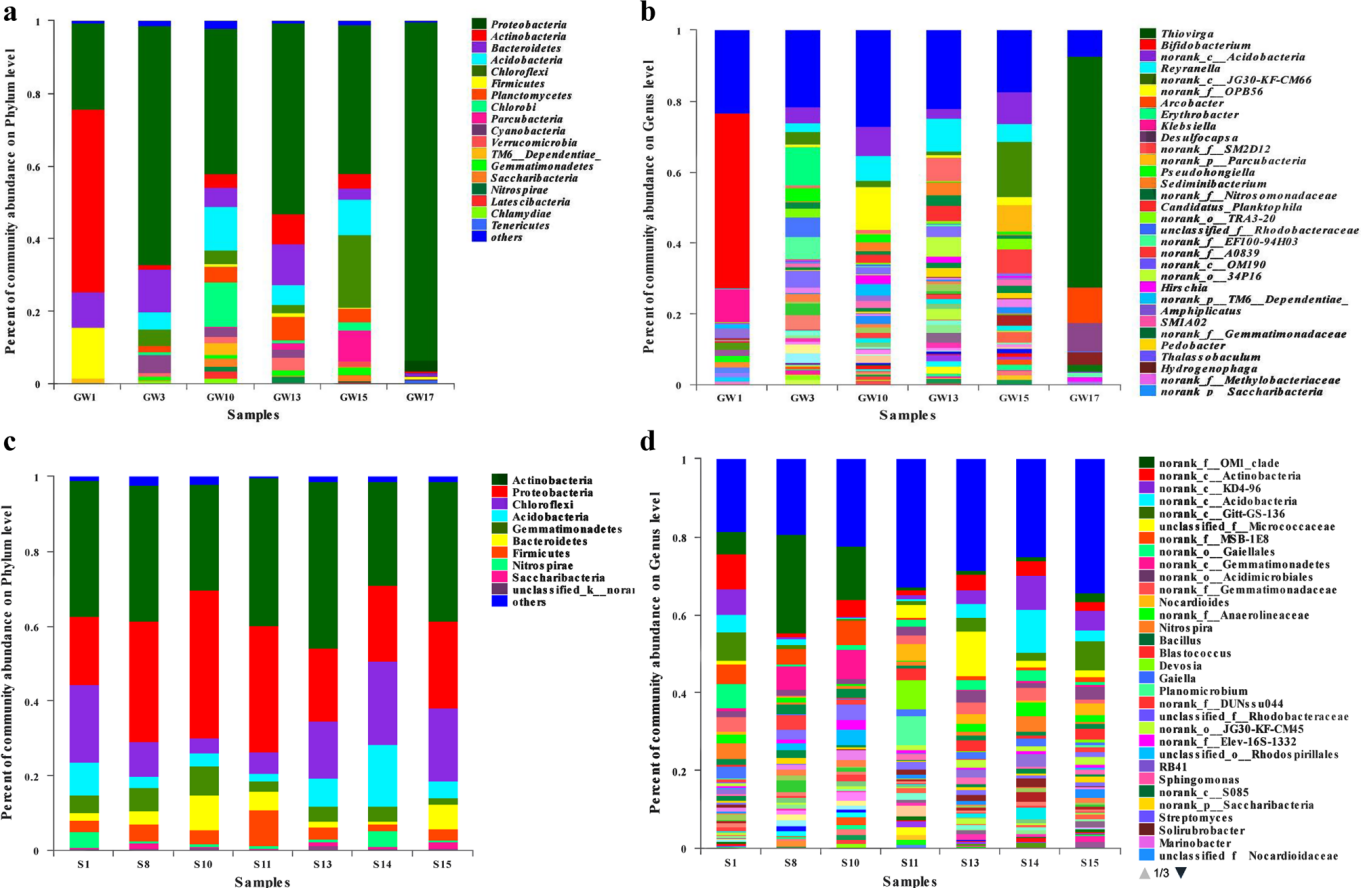
Microbial community diversity in ground water and soil nearby tailings pond. (**a**) Bacterial phylum in ground water (> 1%); (**b**) bacterial genus in ground water (> 1%); (**c**) bacterial phylum in soil (> 1%); (**d**) bacterial genus in soil (> 1%).

At the phylum level, the dominant bacteria in the ground water samples mainly include *Actinobateria*, *Proteobacteria*, *Bacteroidetes*, *Acidobacteria* and *Chlorobi* (Fig. 5a), while in soil samples it is mainly *Actinobacteria*, *Proteobacteria*, *Chloroflexi*, *Acidobacteria*, *Gemmatimonadetes* (Fig. 5c). In the upper reaches, there were single species of microbial community in the ground water, while in the lower reaches, there were greater Microbial diversity, and the dominant bacterial of *Acidobacteria* appeared. That with the distance from the tailings pond farther, the dominant bacterial community of phyla in the soil changed from *Proteobacteria* to *Actinobacteria*. On the contrary, the abundance of *Chloroflexi* increased with the distance increased. At the genus level, *Bifidobacteria* is the dominant bacteria in the upper reaches of tailings ponds, while *Reyranella* is the dominant bacteria in the lower reaches (Fig. 5b). The number of genus with relative abundance indicated that norank bacterial sequences were more abundant in soil samples according the tailings pond (Fig. 5d).

**Figure 6:**
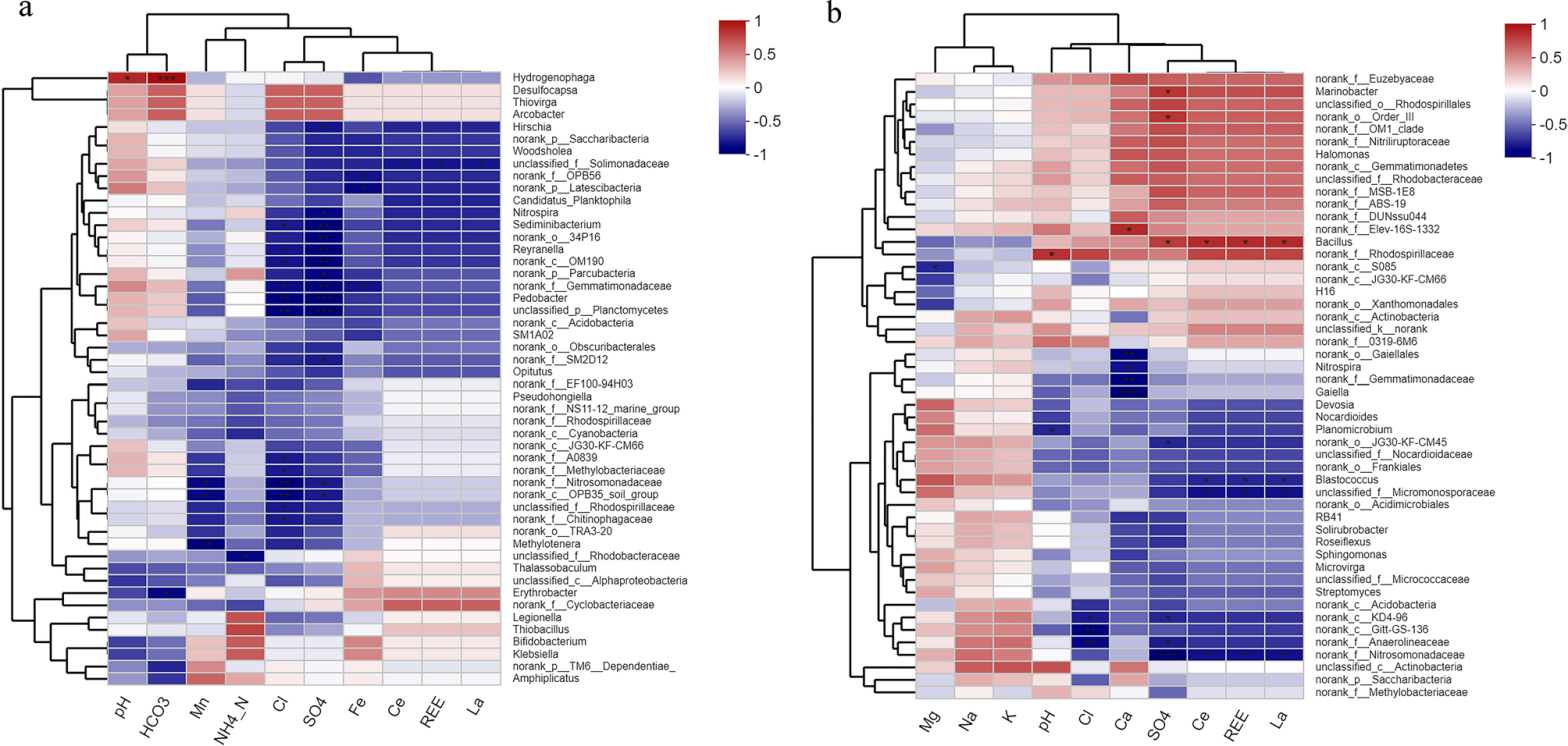
Correlation analysis of microbial communities and environmental factors in ground water (**a**) and soil (**b**) around tailings pond.

The correlation between microbial community and environmental factors was analyzed at genus level. In ground water, *Pedobacter*, *Reyranella* and *Sediminibacterium* were significantly negative correlated with the Cl^−^ and SO_4_^2−^, however, no microbes were found to be significantly associated with ΣREE (Fig. 6a). In soil, *Bacillus* was significantly positive correlated with the SO_4_^2−^, La, Ce and ΣREE, *Blastococcus* also was significantly positive correlated with La, Ce and ΣREE (Fig. 6b).

## Discussion

### The accumulation of rare earth elements in the environment

REEs had an accumulative effect in the environment around the tailings pond. The contents of REEs in the ground water and soil exceeded the background value of Hetao Plane in Inner Mongolia. Hetao Plain in Inner Mongolia is relatively close to the study area and most of the element contents in the area are lower than those in the rest regions of China as well as in the world, which has the similar soil types to the study area^24^. The content of REEs was 4 ~ 10 times higher than that in the ground water from Hetao Plane of Inner Mongolia (the concentration of ΣREE in Inner Mongolia Hetao Plane ground water range from 0.02 ~ 3.45 μg/L)^25^ and 19 times of the background value of soil REEs in Inner Mongolia and mainland China (142.60 mg/kg)^26^.The order of REEs in ground water and soil around the tailings pond from highest to lowest concentration were Ce > La > Nd > Pr > Gd > Sm > Dy > Er > Eu > Yb > Tb > Ho > Tm > Lu, which were similar to that in Bayan Obo ores^27^. Therefore, the accumulation and distribution of REEs in ground water and soil were affected by the leakage after the accumulation of REEs in the tailings pond. Accumulation of REEs in ground water is due to the leakage of the tailings ponds^28^. The main reason for the high content of rare earth elements in the soil around the tailing pond was that the main wind direction in the area is northwest. Wang et al.^29^ found the total concentrations of REEs (ΣREE) for TSP in August 2012 and March 2013 were 172.91 and 297.49 ng/m^3^, respectively. Whereas in the current study the ΣREE in TSP was significantly higher than that in atmospheric particulate matter in Beijing, China and the Netherlands. Due to the flat and open terrain around the tailing pond, the slag stored in the tailing pond spreads to the environment, resulting in a large amount of exogenous rare earth in the soil. Guo et al.^25^ investigated and evaluated the heavy metal pollution in Baotou tailings pond, and reached the same conclusion, holding that the prevailing wind direction in Baotou area northwest wind is the dominant factor affecting the distribution of pollutants.

### Distribution of light rare earth elements around tailings pond

REEs in ground water and soil around the tailings pond all performance as Light rare earth elements enrichment. After the normalization treatment of REEs in the ground water and soil around the tailings pond, it was found that the sampling points near the tailings pond have strikingly similar chondrite-normalized REEs patterns (such as GW3, GW5, GW7, GW7 in Fig. 4a and S5, S7, S8, S10 in Fig. 4b), suggesting that they originated from the same source^30^. But it was also found that the normalized curve was not smooth, this might be due to the presence of REEs in the water mainly in the form of complexes or particulate matter. As REEs are generally insoluble in water the concentration was low, so a regular figure was not presented^31^. The LREEs/HREEs ratio was usually considered as an indicator to measure LREEs and HREEs fractionation^32^. The ratios of (La/Sm)_N_ and (Gd/Yb)_N_ were used to measure the degree of LREEs fractionation and HREEs fractionation, respectively. According to the results of LREEs/HREEs, (La/Sm)_N_ and (Gd/Yb)_N_, both the groundwater and the soil showed light rare earth enrichment. Our findings were consistent with a range of previous studies that LREE enrichment relative to HREE particularly from the analysis of sediment core samples taken from a fairly polluted marine environment, in the sediment samples^33^, and soils developed on four geological units (tropical Terengganu River Basin, Malaysia)^34^. While, in the vicinity of a large-scale mining site in southwest Fujian Province, China, the distribution patterns of REEs in soil samples were characterized by LREEs enrichment, but HREEs enrichment in water sample^35^. The fractionation patterns of LREEs enrichment might be due to the reductive dissolution of ferromanganese oxide, which results in release of preferentially scavenged LREEs into ground water^36^. Moreover, LREEs accounted for more than 73% of the total ground water REEs burden. Such distribution patterns of LREEs enrichment were similar to those in Bayan Obo ores^27^. In addition, the more exogenous REEs from the slag, the higher the ratio of LREEs/HREEs, which was the reason for the high content of LREEs in the soil samples^37^.

Different extents of anomaly (from negative to positive) were observed for Ce and Eu in the distribution patterns of REEs in groundwater. However, Ce and Eu were negatively anomaly in soil. Ce is often oxidized from the soluble trivalent state to the insoluble tetravalent state in water. Ce^4+^ preferentially combines with the Fe–Mn oxides on the particulates to enter the particulate phase, or precipitates from the water in the form of CeO_2_, leading to negative Ce anomaly in groundwater. The positive Ce anomaly in groundwater may be caused by the re-release of the absorbed Ce after the dissolution of Fe–Mn oxide in the reducing environment, but the correlation between Ce, Mn^2+^ and Fe was not significant, indicating that the positive Ce anomaly was also controlled by other processes^38–40^. And compared with Eu, the content of Sr in groundwater was very high. Due to the similar chemical properties of Eu^2+^and Sr^2+^, Eu^2+^ and Sr^2+^ exchange might lead to Eu positive anomaly^38^. In addition, the decomposition of feldspar minerals might also lead to positive Eu anomalies^40,41^. Both Ce and Eu showed negative anomalies in the soil, which were related to the parent rocks in the study area^42,43^.

### Interaction between rare earth elements and chemical properties

In ground water, correlation analysis between REEs, δCe, δEu and physical–chemical indexes showed that the content of REEs was directly or indirectly affected by pH, adsorption or complexation of iron and manganese oxides and redox conditions in ground water. REEs, to some extent, was related to Mn^2+^. Mn^2+^ and sulfate form a manganese sulfate complex that led to an increase of manganese ion concentration, and the adsorption or complexation of REEs by hydroxides or oxides of ferromanganese. This led to the change of rare earth element concentration in ground water^39^. It was generally believed that the pH can affect the REEs adsorption and complexation and control of ground water concentration of REEs^44–46^. Nevertheless, in our study there was no obvious correlation between the total REEs concentration and pH, which was similar to the ground water from the Hetao Plane of Inner Mongolia. REEs in the deposit mainly occur in the granite weathering layer and are generally adsorbed in soil/sediments as ions. Currently, the advanced in situ leaching method was widely used to separate and extract ion-adsorbed REEs in China^47^. While this was an effective method for the REEs exploitation, chemicals such as ammonium sulfate or ammonium bicarbonate need to be injected into the soil/sediment to extract rare earth. Therefore, the concentration of ammonia nitrogen in tailings and wastewater was relatively high, and we inferred that a part of ammonia nitrogen in the tailings has been transferred into aquatic environments by rain-wash or leakage^48^. This might be the reason for increasing of the REEs, which was saw associated with ammonia nitrogen. However, the correlation analysis between REEs and physicochemical properties in soil showed that Ca^2+^ was positively correlated with La, Ce and REEs, Ca^2+^ was one of the main ions exchanged by soil particles that non-obligate adsorption of exogenous rare earth, which leads the much greater increase of Ca^2+^ than other ions^49^, and the correlation between SO_4_^2−^, Cl^−^ and REEs was due to the influence of human input. Therefore, it was of great significance to control the pollution of the tailings pond by controlling the input of artificial sources and taking quick measures.

### Correlation between rare earth elements and dominant bacteria

By using a high-throughput sequencing platform, the typical ground water and soil samples around the tailings pond were sequence. The dominant bacteria in soil and ground water are consistent. The dominant bacteria were *Actinobateria*, *Proteobacteria* and *Acidobacteria* at phylum level. This microbial community composition is similar to that reported in arid lands around the world^50,51^. *Actinobateria* is highly resistant to drought and low resource conditions, which may make them superior to other microbial groups under extreme conditions^52^. Regardless of the age of the species, *Proteobacteria* and *Acidobacteria* are the most abundant phyla, usually consistent with other findings, that is, the ground water and soil usually contains two ubiquitous bacterial groups^53,54^. Li et al.^55^ reported similar soil restoration findings in the time series of 1–20 years after the cessation of mining operations. This finding suggests that, among other factors, nutrient restriction favors *Proteobacteria*, which may play a functional role in soil recovery for decades. Many *Proteobacteria* are symbiotic bacteria that can become rich once the substrate is available, while *Acidobacteria* prefer to live in undernourished environments^56^. The bacterial communities in our study generally changed from *Proteobacteria* to *Acidobacteria* in soil and *Acidobacteria* appear downstream from ground water, may be due to the pollution of tailings pond, which creates an environment suitable for the growth of *Acidobacteria*. On the genus level, *Reyranella* is the dominant bacteria in ground water. Nitrogen source of *Reyranella* is usually ammonium salt and a little organic nitrogen^57^. The leakage of tailings pond increases the content of ammonia nitrogen in ground water and provides nitrogen source for *Reyranella*. REEs are positively correlated with *Bacillus* and negatively correlated with *Blastococcus*. *Bacillus* has a variety of physiologic abilities that allow it to live in a wide range of habitats, including many extreme habitats such as desert sand, hot springs and arctic soil. In harsh environments such as low temperature, high heat and radiation, *bacillus* will release spores to resist extreme environments^58^. This may explain the positive correlation between *Bacillus* and REE in environments with large amounts of rare earths. However, their directive function should be verified by experimental evidence in future.

## Conclusion

The tailings pond was observed to be a considerable rare earth elements (REEs) anthropogenic sources. REEs around the tailings showed a remarkable concentration gradient, and the concentration surrounding tailings pond was much higher than that of other sampling points. The rare earth distribution patterns of ground water around the tailings pond were enriched in LREEs and different extents of anomaly (from negative to positive) were observed for Ce and Eu in the distribution patterns of REEs in groundwater. However, Ce and Eu were negatively anomaly in soil. Correlation analysis between REEs and chemical indexes showed the main chemical indexes affecting REEs in ground water were Mn^2+^ and ammonia nitrogen, while in soil it changes to Ca^2+^, SO_4_^2−^ and Cl^−^. The dominant bacteria in ground water and soil at the phylum level were *Actinobateria*, *Proteobacteria* and *Acidobacteria*. It is feasible to use *Bacillus* and *Blastococcus* as indicators of REEs. The results indicated that REEs emission from human activities will accumulate in ground water and soil. Although the content is low, it will certainly cause environmental pollution over time. This study might be of guided significance for the artificial source of REEs.

## Supplementary information

Supplementary information
