# Supplementary material for: Geobiochemistry characteristics of rare earth elements in soil and ground water: a case study in Baotou, China
Source: Sci Rep. 2020 Jul 16;10:11740. doi: 10.1038/s41598-020-68661-4 (PMC7367286; doi:10.1038/s41598-020-68661-4)
Supplement: Supplementary file 1 — Supplementary information [file 41598_2020_68661_MOESM1_ESM.docx]

**Supplementary material**

**[Geobiochemistry](javascript:;) characteristics of rare earth elements in soil and ground water: A case study in baotou, China**

Shuting Tang^a^, Chunli Zheng^a*^, Minjie Chen^b^, Weiqi Du^a^, Xin Xu^a^

^a^School of Energy and Environment, Inner Mongolia University of Science and Technology, Baotou 014010, People’s Republic of China

^b^School of life science and technology, Inner Mongolia University of Science and Technology, Baotou 014010, People’s Republic of China

* Correspondence: Chunli Zheng, Tel: +86-0472-5952278, Fax: +86-0472-5951567,

**Email:** [**nm_wx@163.com**](mailto:nm_wx@163.com)


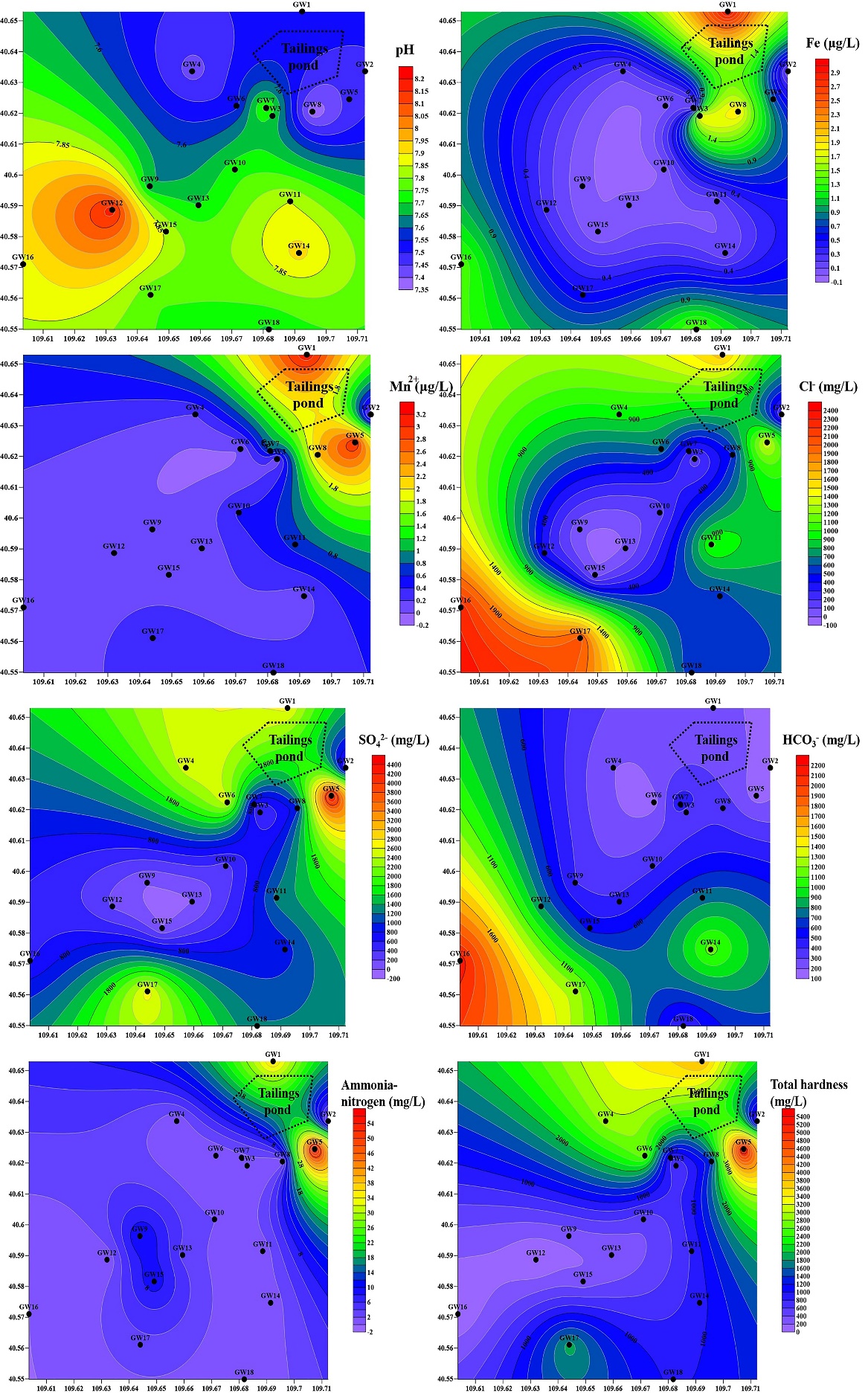
**Figure S1.** Basic chemical properties of the sampling points in ground water nearby tailings pond. Fe, Mn^2+^, Cl^-^, SO_4_^2-^, ammonia nitrogen and total hardness shown the same trend and decreased with distance


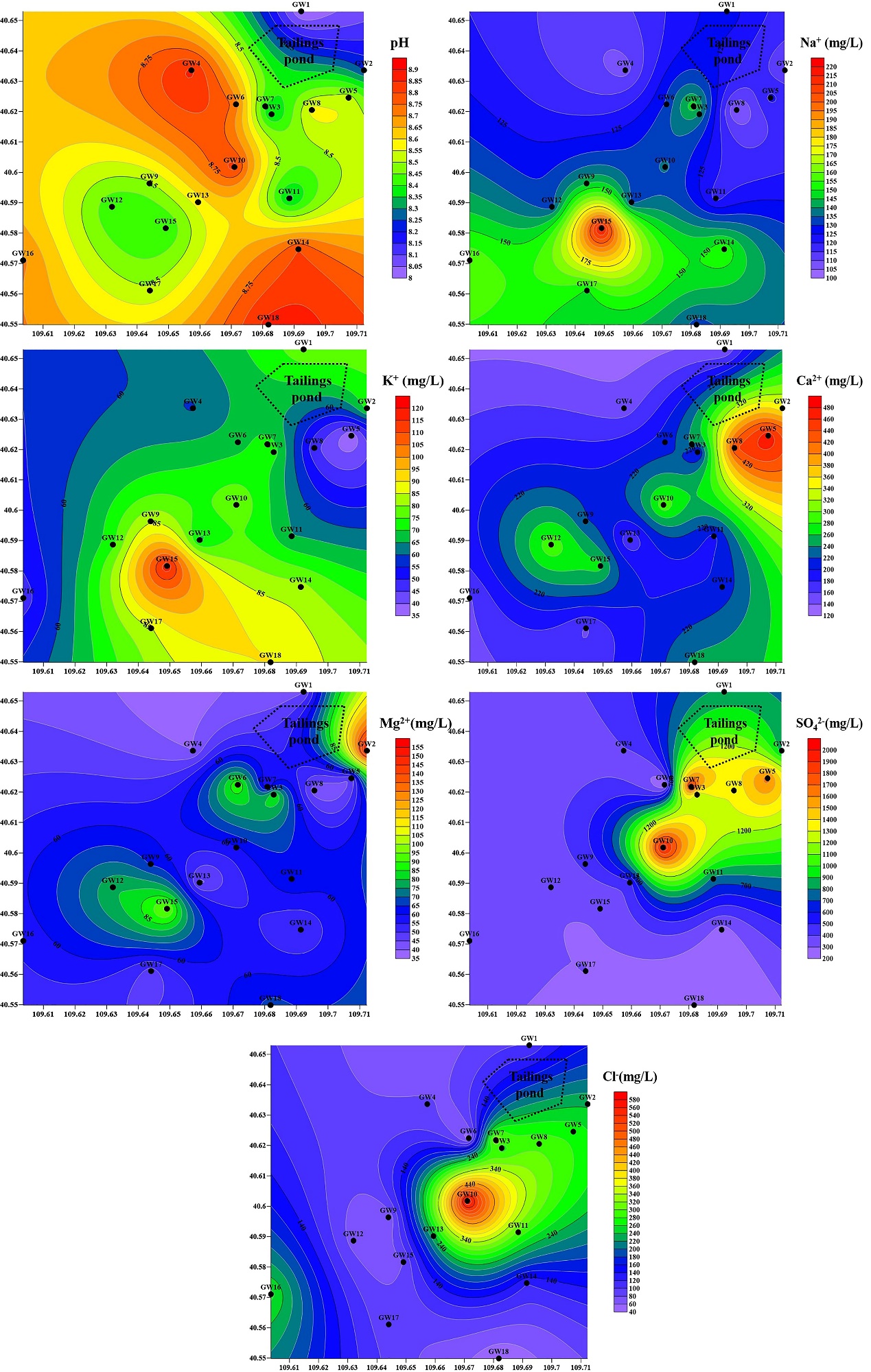


**Figure S2.** Basic chemical properties of the sampling points in soil nearby tailings pond. The contents of SO_4_^2-^ and Cl^-^ decreased with the increase of the distance from tailings pond, while Na^+^, K^+^, Ca^2+^ and Mg^2+^ showed no significant change trend

Table S1. The Alpha diversity index (Shannon index)

|  | Sample | Shannon |
| --- | --- | --- |
| Soil | S1 | 5.747647 |
|  | S8 | 5.200799 |
|  | S10 | 5.176408 |
|  | S11 | 5.532887 |
|  | S13 | 5.824894 |
|  | S14 | 6.011548 |
|  | S15 | 5.910690 |
| Ground water | GW1 | 2.919978 |
|  | GW3 | 4.572700 |
|  | GW10 | 4.626175 |
|  | GW13 | 4.615983 |
|  | GW15 | 4.547426 |
|  | GW17 | 1.577382 |
